# “Causes of death in asthma, COPD and non-respiratory hospitalized patients: a multicentric study”

**DOI:** 10.1186/1471-2466-13-73

**Published:** 2013-12-10

**Authors:** Jose Gregorio Soto-Campos, Vicente Plaza, Joan B Soriano, Carlos Cabrera-López, Carlos Almonacid-Sánchez, Rosa Vazquez-Oliva, Jose Serrano, Aitor Ballaz-Quincoces, Alicia Padilla-Galo, Vanessa Santos

**Affiliations:** 1 Unidad de Gestion Clínica de Neumología y Alergia, Hospital de Jerez, C/ Sevilla n° 42; 1° J. C.P, Cádiz 11402, Spain; 2 Hospital de la Santa Creu i Sant Pau, Barcelona, Spain; 3 Programa de Epidemiología e Investigación Clínica, Fundación Caubet-CIMERA, Illes Balears, Spain; 4 Hospital Universitario de Gran Canaria Dr. Negrín, Las Palmas de Gran Canaria, Spain; 5 Hospital de Guadalajara, Guadalajara, Spain; 6 Hospital Infanta Elena, Huelva, Spain; 7 Hospital Comarcal de Inca, Palma de Mallorca, Spain; 8 Hospital de Galdakao, Vizcaya, Spain; 9 Hospital Costa del Sol, Málaga, Spain; 10 Fundación para la Gestión de la Investigación Biomédica de Cádiz, Cádiz, Spain

**Keywords:** Asthma, COPD, Respiratory failure, Mortality, Cardiovascular diseases.

## Abstract

**Background:**

There is limited information on the causes of death in asthma patients.

To determine the causes of death in hospitalized asthmatic patients and to compare with those observed in COPD patients and non-respiratory individuals, with a particular interest in associations with previous cardiovascular disease.

**Methods:**

Retrospective case–control study which analyzed the deaths of all hospitalized patients admitted for any reason during January, April, July and October of 2008 in 13 Spanish centers. Medical records of deceased patients were reviewed, and demographic and clinical data were collected.

**Results:**

A total of 2,826 deaths (mean age 75 years, 56% men) were included in the analysis, of which 82 (2.9%) were of patients with asthma and 283 (10%) with COPD.

The most common causes of death in asthma patients were cardiovascular diseases (29.3%), malignancies (20.7%) and infections (14.6%); in COPD patients they were malignancies (26.5%), acute respiratory failure (25.8%) and cardiovascular diseases (21.6%). Asthma, compared to COPD patients, died significantly less frequently from acute respiratory failure and lung cancer. A multivariate logistic regression analysis failed to associate asthma with cardiovascular deaths.

**Conclusions:**

Cardiovascular disease is the most frequent cause of death among hospitalized asthma patients. The specific causes of death differ between asthma and COPD patients.

## Background

After the epidemics of asthma deaths during the 1990s, numerous International [1,2] and national [3-5] recent studies have confirmed a consistent decrease in mortality. Among the causes of this decline, the correct implementation of clinical practice guidelines has been postulated [6-8], and particularly, the increasingly universal use of inhaled corticosteroids as maintenance asthma treatment [9,10].

Unlike COPD, for which mortality has risen during the same period [11], the reduced mortality from asthma has perhaps contributed to a reduced knowledge on the clinical characteristics and circumstances of death in asthmatics. Thus, contrary to COPD patients, in whom numerous recent studies have characterized their causes of death, in asthma patients there has been considerably less research. It is well established that in COPD, serious exacerbations, respiratory failure, cardiovascular diseases, and bronchopulmonary malignancies constitute the principal causes of death [12-14]. Variation in the relative importance of these causes according to the severity of COPD has also been described as lung cancer is the principal cause of death in mild COPD, while respiratory failure is in very severe COPD [15].

In asthma, the possible causes of death are still not well understood, except for the recognized reduced contribution of severe asthma exacerbations. The few studies available are limited to those designed with other epidemiological objectives [16]; others have been conducted in specific population groups (women [17], children [18]), or are based on series of deaths too small to draw strong conclusions [19].

Furthermore, the bronchial and systemic inflammation and the clinical impact of the associated comorbidities in asthma [20-23] have provided evidence for suspecting that, as in COPD [12,24], asthma patients might be at a greater risk of suffering cardiovascular [25,26] and cerebrovascular [27] diseases and, therefore at risk of death due to vascular events [17,19]. However, previous studies exploring this association have reported conflicting results, and few of these have managed to determine a causal relationship between asthma and cardiovascular disease. Also, the method used in some of these studies could be questioned. For example, the diagnosis of asthma was assumed from simple self-reporting by patients [19,25-27]; and, in others, by their responses collected in a telephonic survey addressed to recognize asthma symptoms [17].

Our study was designed with two objectives: to determine the causes of death of patients with asthma compared with those of patients with COPD; and to establish a possible relationship between deaths from asthma and cardiovascular diseases. In Spain about 68% of deaths occur in hospitals with certain regional geographic variation [28]. We believe that the information collected is representative because most of the Spanish population deaths occur in hospitals.

## Methods

Observational, retrospective, cross-sectional, multi-centre study conducted in a large group of patients who died while admitted in hospital.

Fieldwork included all deaths in admitted patients during the months of January, April, July and October (months taken as representative of each of the four seasons) of 2008 in thirteen Spanish secondary and tertiary hospitals. The clinical records of all deceased patients were reviewed, and the following data were collected: demographic data; existence of risk factors for cardiovascular diseases (smoking, diabetes, arterial hypertension, obesity, hyperlipidaemia); and specific causes of death, distinguishing specifically lung cancer within neoplastic disorders, the pneumonia within infections; and within cardiovascular disease, peripheral arteriopathy, ischemic cardiopathy, cerebrovascular disease or heart failure. In addition, in patients with asthma, severity and control (both by GINA [6] and GEMA [8] criteria); the frequency of scheduled and unscheduled medical visits, spirometry (best FEV1 recorded) and prescribed treatment were also recorded for each death. The study protocol was approved by a central ethics committee (Hospital of Jerez) following the rules of the Helsinki Declaration.

Previous asthma diagnosis was considered of a high degree of certainty, when the clinical record contained diagnostic confirmatory reports (signed by a pulmonologist or allergy specialist) with objective tests like either significant variability of the spirometric flows performed by applying a bronchodilatator or bronchoconstriction challenge test; alternatively, a label of moderate degree of certainty was given in absence of the above mentioned criteria and any of the following: the patient clinical record reported symptoms compatible with asthma, prescription of asthma drugs or an indication that the attending physician during the hospital admission presumed the asthma diagnosis. Indistinguishable cases from COPD, bronchiectasis or residual pulmonary lesions after tuberculosis co-existed, were excluded from the analysis. A diagnosis of COPD was considered when there was a diagnostic medical report (signed by a pulmonologist) supported by poorly reversible airflow limitation with a post-bronchodilator FEV1/FVC ratio < 0.70 ( GOLD criteria) [29].

### Statistical analysis

A descriptive analysis was carried out for all variables collected. Summary results were expressed as percentages, frequencies and number of observations for qualitative variables, and as means with standard deviation for quantitative variables.

We check the normality or otherwise of the sample using the Kolmogorov Smirnov. Comparisons between groups were performed using the χ 2 test for categorical variables; and for quantitative variables, the ANOVA test, or the Kruskall-Wallis test if values did not have a normal assumption. For control of type I error rate, we penalized or adjusted p values for multiple comparisons. A multivariate logistic regression analysis was performed to determine the possible independent association of a previous diagnosis of asthma with death due to cardiovascular disease; this latter was considered both globally and stratified by four predetermined categories (peripheral arterial disease, ischemic cardiopathy, cerebrovascular disease, and heart failure). The dependent variable was death by cardiovascular diseases and the independent variables, a previous diagnosis of asthma or COPD, age, sex, smoking, previous history of diabetes mellitus, obesity, hyperlipidaemia, and arterial hypertension. Comparisons with a p value <0.05 were considered statistically significant.

## Results

The cause of death was determined according to certification that existed in medical history signed by the doctor who treated the patient, no death was excluded. A total of 2,826 deaths were included in the analysis. Of these, 1,583 (56%) were men. The mean ± SD age was 75 ± 14 years (74 ± 14 in men vs 77 ± 15 in women, p = 0.01). The most frequent causes of death observed in the total sample were: 746 (27%) malignancies, of which 173 (23%) were lung cancer; 710 (25.7%) cardiovascular diseases (258 heart failure, 208 cerebrovascular disease, 191 ischemic cardiopathy, and 53 peripheral arterial disease); and 597 infections (21%), of which 332 (54.6%) pneumonia. Farther 251 deaths were observed gastrointestinal origin (9%) and 206 from respiratory failure (72 COPD patients, 5 asthmatics and 131 from other causes and unclear diagnoses).

Table 1 presents and compares the demographic and clinical characteristics of the sample distributed in, COPD (283 [10%]), asthma (82 [2.9%]) and neither of these two diseases (2,461 [87%]) groups. Patients with COPD were more frequently male and smokers, while those with asthma were more frequently obese (p < 0.001). The overall comparison of the three groups using ANOVA showed significant differences in gender (p < 0.001), smoking (p < 0.001), obesity (p = 0.002) and BMI (p < 0.001).

**Table 1:** Demographic and clinical characteristic in the 2,826 deaths analyzed, by diagnosed asthma, COPD or neither of the two diseases (without asthma/COPD)

	**Asthma (n = 82)**	**COPD (n = 283)**	**Without asthma/COPD (n = 2.461)**	**Value of *p***
				0.350^*^
**Age** (years), mean (SD)	75 (15)	76 (9)	75 (14)	0.690^†^
CI 95%	(60–80)	(68–85)	(61–89)	0.310^¶^
**Sex** n(%)				
– Men	18 (22)	256 (90.4)	1280 (53.0)	< 0.001^*^
– Women	64 (78)	27 ( 9.6)	1171 (47.0)	**< 0.001**^†^
				**< 0.001**^¶^
**Smoking** n (%)				
– Smoker	8 (10.5)	64 (22.7)	295 (13.8)	< 0.001^*^
– Former smoker	9 (11.8)	208 (73.6)	547 (25.5)	0.008^†^
– Non smoker	58 (77.7)	10 (3.7)	1300 (60.7)	**< 0.001**^¶^
**Diabetes** n (%)				0.266^*^
	28 (34.6)	77 (27.7)	652 (26.9)	0.081^†^
				0.417^¶^
**Hyperlipidaemia** n (%)				0.035^*^
	26 (32.1)	58 (21.5)	590 (24.8)	0.090^†^
				0.121^¶^
**AHT** n (%)				0.148^*^
	43 (52.8)	184 (65.0)	1321 (53.7)	0.529^†^
				0.030^¶^
**Obesity** BMI > 30 kg/m^2^ n (%)				0.033^*^
	27 (32.8)	57 (20.1)	339 (13.8)	**< 0.001**^**†**^
				0.011^¶^
**BMI,** mean (SD) CI 95%				0.061^*^
	27.6 (7)	25.2 (5.4)	23.7 (6)	**< 0.001**^†^
	(20,6-34,6)	(19,8-30,6)	(17,7-29,7)	**0.002**^¶^

Footnote:

Values represent means (standard deviation) or number of cases (percentage).

*AHT*, arterial hypertension; *BMI*, body mass index.

*comparisons between asthma group and COPD group

†comparisons between asthma group and group with neither asthma nor COPD.

¶comparisons between COPD group and group with neither asthma nor COPD.

comparisons between groups using the χ^2^ test for the categoric variables; and for the ordinal or quantitative variables, using the ANOVA test, or the Kruskall-Wallis test if the values did not present a normal distribution.

We considered significanta p-value < 0.005.

The most frequent causes of death in asthmatics (Table 2) were cardiovascular diseases (29.3%), malignancies (20.7%) and infections (14.6%); in COPD patients, causes of death were malignancies (26.6%, of which 54% was broncho-pulmonary), acute respiratory failure (25.5%) and cardiovascular diseases (21.6%). It is worth noting that the proportion of patients who died from pneumonia in the COPD group (59% of all fatal infections), was greater than in the asthma group (39%), but this difference did not reach statistical significance (p = 0.15). Only five (6%) deaths due to respiratory failure (exacerbation) were documented in the asthma group, while 72 (25.5%) occurred in patients with COPD (p < 0.05). The comparison of the causes of mortality among the three groups using ANOVA showed significant differences in solid malignancies (p < 0.001), respiratory insufficiency (p < 0.001) and other causes (p = 0.008).

**Table 2:** Causes of death in the 2,826 deaths analyzed, by diagnosed asthma, COPD or neither of the two diseases (without asthma/COPD)

**Causes of death**	**Asthma (n = 82)**	**COPD (n = 283)**	**Without asthma/COPD (n = 2.461)**	**Value of *p***
**Solid malignancies,** n (%)	17 (20.7)	75 (26.6)	654 (26.6)	< 0.001^*^
– Pulmonary, n (%)	1 (1.2)	41 (14.5)	131 (5.3)	0.088^†^
				**< 0.001**^¶^
**Cardiovascular diseases,** n (%)	24 (29.3)	61 (21.6)	641 (26.1)	
– Ischemic cardiopathy, n (%)	6 (7.3)	16 (5.6)	169 (7.0)	0.100^*^
– Cerebrovascular accident, n (%)	6 (7.3)	19 (6.7)	183 (7.6)	0.394^†^
– Peripheral arteriopathy, n(%)	0 (0.0)	9 (3.2)	44 (1.8)	0.136^¶^
– Heart failure, n (%)	12 (14.6)	17 (6.0)	229 (9.5)	
**Infections,** n (%)	12 (14.6)	46 (16.3)	539 (21.9)	0.154^*^
– Pneumonias n (%)	5 (6.0)	29 (10.3)	298 (12.1)	0.192^†^
				0.320^¶^
**Digestive diseases,** n (%)				< 0.001^*^
	7 (8.5)	19 (6.7)	225 (9.2)	**< 0.001**^†^
				**< 0.001**^¶^
**Respiratory insufficiency,** n (%)				< 0.001^*^
	5.0 (6)	72 (25.5)	122 (4.9)	**< 0.001**^†^
				**< 0.001**^¶^
**Other♯,** n (%)				< 0.001^*^
	17 (20.9)	9 (3.3)	275 (11.3)	**< 0.001**^†^
				**< 0.001**^¶^

Footnote:

Values represent means (standard deviation) or number of cases (percentage).

Includes deaths from diseases of the blood and hematopoietic organs, diseases of the Central Nervous System, endocrine diseases, pulmonary thrombo-embolism, diseases of the connective tissue, renal insufficiency, and accidents.

*comparisons between asthma group and COPD group.

†comparisons between asthma group and group with neither asthma nor COPD.

¶comparisons between COPD group and group with neither asthma nor COPD.

Comparisons between groups using the χ^2^ test for the categoric variables; and for the ordinal or quantitative variables, using the ANOVA test, or the Kruskall-Wallis test if the values did not present a normal distribution.

We considered significanta p-value < 0.005.

The clinical and functional respiratory characteristics of cases with asthma are shown in Table 3 (differences between asthma high certainty and asthma lower certainty). Notable among these results are the small proportion of patients with asthma well controlled (14.5%), the high frequency of moderate or severe persistent asthma (49%), and the high frequency of patients with a history of hospital admission for an asthma exacerbation (48%). As well the low rates of preventative treatment received by patients: 16.9% without treatment; 14.1% only with a short acting β2-adrenergic agonist on rescue medication; and 2.8% with a long-acting β2-adrenergic agonist without inhaled corticosteroids.

**Table 3:** Previous clinical and functional respiratory characteristics of all patients with asthma who died, by the degree of certainty of the diagnosis of asthma

	**All (n = 82)**	Asthma high certainty^* ^**(n = 26)**	Asthma lower certainty^* ^**(n = 56)**	**Value of *p***
**Age** mean (SD)	75 (15)	70 (15)	78 (15)	0.061
**Sex**				
– **men**	52	23.1	21.4	0.869
– **women**	47	76.9	78.6	
**BMI** mean (SD)	23.7 (6)	27.8 (7.2)	27.5 (7.1)	0.900
**Hospitalizations due to asthma**. n (%)				
– Patients admitted	26 (48.1)	10 (38.5%)	16 (28.6%)	0.838
– Number of admissions per patient	2.03 (1.8)	2.83 (2.3)	1.55 (1.3)	
**Asthma control (GINA),** n (%)				
Uncontrolled	41 (59.4)	14 (58.3)	27 (60)	0.791
Partly controlled	18 (26.1)	6 (25.0)	12 (26.7)	
Controlled	10 (14.5)	4 (16.7)	6 (13.3)	
**GINA severity,** n (%)				
Intermittent,	17 (28.8)	3 (14.3)	14 (36.8)	0.075
Persistent, mild	13 (22.0)	2 (9.5)	11 (28.9)	
Persistent, moderate	21 (35.4)	11 (52.4)	10 (26.4)	
Persistent, severe	8 (13.6)	5 (23.8)	3 (7.9)	
**Spirometry,** mean (SD)				
– FEV_1_, ml	1.215 (767)	1453 (739)	907 (709)	0.692
– FEV_1_,%	64.4 (23)	63.2(22.4)	66.5 (25.2)	
**Previous maintenance treatment,** n (%)				
– LABA + ICS	19 (26.7)	12 (46.1)	7 (15.6)	0.122
– LABA + ICS + LTRAs	13 (18.3)	8 (30.8)	5 (11.1)	
– No treatment	12 (16.9)	3 (11.5)	9 (20)	
– Short-acting β_2_-adrenergic agonist	10 (14.1)	2 (7.8)	8 (17.8)	
– LTRAs	9 (12.7)	0	9 (20)	
– LABA+ICS+oral corticosteroids	3 (4.2)	0	3 (6.7)	
– Other:				
– Ipratropium bromide	2 (2.8)	0	2 (4.4)	
– LABA only	2 (2.8)	1 (3.8)	1 (2.2)	
– ICS only	1 (1.4)	0	1 (2.2)	

Footnote: Values represent means (standard deviation) or number of cases (percentage).

*FEV*_*1*_, forced expiratory volume in the first second; *ICS*, inhaled corticosteroids; *LABA*, long-acting β2-adrenergic agonists; *LTRAs*, leukotriene receptor antagonists.

^*^Comparisons between groups were not significant using the χ^2^ test for the categoric variables; and for the ordinal or quantitative variables, using the ANOVA test, or the Kruskall-Wallis test if the values did not present a normal distribution.

Differences between asthma high certainty and asthma lower certainty.

The results of the multivariate analysis to determine the risk factors associated with death by cardiovascular diseases, are shown in Table 4. It is reported an association with the well-known classical cardiovascular risk factors, namely diabetes, age, hyperlipidaemia, but there was no association with a previous asthma diagnosis. When ‘death due to cardiovascular disease’ was stratified into the four categories considered independently (cerebrovascular disease, ischemic cardiopathy, peripheral arterial disease and heart failure), no association was found with a previous asthma diagnosis as well; OR 0.85 (95% CI 0.36 to 2.01) p = 0.74 for cerebrovascular disease; 0.86 (95% CI 0.33 to 2.21) p = 0.76 for ischemic cardiopathy; undetermined by peripheral arterial disease and OR 1.57 (95% CI 0.83 to 2.99) p = 0.16 for heart failure (see Table 5).

**Table 4:** Multivariate analysis of factors associated with death due to cardiovascular diseases (crude and adjusted rates)

	**n**	**Odds ratio crude**	**CI 95% (OR crude)**	**Odds ratio adjusted**	**CI 95% (OR adjusted)**
**Asthma - high certainty**	26	0.69	0.26 - 1.82	0.64	0.23 - 1.77
**Asthma - probable**	56	1.49	0.85 - 2.62	1.1	0.60 - 2.01
**COPD**	283	0.775	0.58 - 1.04	0.84	0.60 - 1.18
**Former smoker**	764	0.88	0.73 - 1.07	0.96	0.74 - 1.24
**Smoker**	367	0.6	0.45 - 0.79	0.93	0.67 - 1.30
**Age**	2826	1.03	1.02 - 1.04	1.02	1.02 - 1.03
**Sex**	2816	0.707	0.60 - 0.84	0.83	0.66 - 1.03
**Hyperlipidaemia**	674	2.01	1.67 - 2.43	1.82	1.49 - 2.22
**Diabetes**	757	1.87	1.56 - 2.25	1.57	1.29 - 1.90

Footnote: *95% CI*, 95% confidence interval *OR*, odds ratio.

**Table 5:** Results of the multivariate analysis performed to determine the possible association of the previous diagnosis of asthma with causes of death from heart failure

	**Odds ratio**	**CI 95%**	**P-value**
**Asthma**	1.57	0.83-2.99	0.16
**Diabetes**	1.20	0.89-1.61	0.21
**Former smoker**	0.79	0.53-1.16	0.23
**Smoker**	0.55	0.30 - 1.02	0.05
**Age**	1.02	1.01 - 1.03	<0.001
**Sex**	0.79	0.57 - 1.11	0.18
**Hyperlipidaemia**	1.07	0.82 - 1.41	0.58
**AHT**	1.75	1.13 - 2.69	<0.001

Footnote:

*CI 95%*, confidence interval at 95%.

*AHT*, arterial hypertension.

The main results were not substantially modified when asthmatics were divided according to the degree of certainty of the asthma diagnosis.

## Discussion

The main finding of the present study was that cardiovascular diseases are the most frequent cause of death in patients with asthma. And compared with COPD patients, deaths due to exacerbations and malignancies were less frequent. These findings are particularly relevant given the limited information on this topic.

Our study was designed to include deaths of patients with asthma of any age and both sexes; consequently it probably is more representative to the total population of asthmatics than previous studies carried out in specific sub-groups. For instance, de Marco et al19 evaluated the causes of death of 47 asthmatics aged 20 to 44 years, and found that 34% died due to malignancies and 17% of accidents. Similarly, Camargo et al [17] analyzed deaths of 87 women with asthma (all nurses) aged 30 to 55 years, of whom 31% of deaths were malignancies and 22% cardiovascular diseases.

Another key results from our study comes from the inclusion of deaths of COPD patients. Whereas in asthma cardiovascular diseases constitute the most frequent cause (29.3%), malignancies were the most frequent cause (26.5%) in COPD, likely associated with a higher cumulative tobacco exposure. Similarly, patients with asthma died less frequently than patients with COPD of respiratory failure or during exacerbation of their disease (6.0% vs. 25.5%), due to lung cancer (1.2% against 14.5%) and due to pneumonia (6% against 10.3%). The finding that patients with asthma, in contrast to COPD patients, die relatively infrequently from an exacerbation of their disease, was well-documented in several earlier series. Silverstein et al [16] found in a cohort of 2,499 persons with asthma that only 4% of the 140 deaths recorded during 14 years of follow-up were by an episode of exacerbation. This frequency is very similar (4.6%) out from 87 deaths reported by Camargo et al [17]. Contrary in COPD, serious worsening constitutes one of the most frequent causes of death. In the TORCH study [12] this was determined as the cause of 35% of deaths in 875 deceased COPD patients. Similarly, lung cancer constitutes a frequent cause of death in COPD: 21% in the TORCH study [12], and 38% in the Lung Health Study [13]; as above mentioned, it is not a frequent cause of death in asthma. Finally in terms of differences in the frequency of pneumonia as cause of death in these two diseases. It had been suggested that patients with COPD treated with inhaled corticosteroids have a greater risk of contracting pneumonia, but do not die of it [12,30], and some authors have suggested a potential beneficial effect, or at least not a deleterious effect, of the outpatient use of inhaled corticosteroid in COPD or asthmatics patients who developed pneumonia [31,32]. In our series, there is a considerable percentage of patients with COPD who died of pneumonia, but not higher than in the group of patients without previous lung pathology or asthmatics patients.

Paradoxically, since patients with asthma are nowadays more likely to survive with close to normal longevity, it resulted in an increase in prevalence of certain comorbidities associated with the disease having a significant impact on their quality of life and probably also on their specific causes of death [33]. Among others, the cardiovascular diseases are significant, and in particular, cardiac disease and cerebrovascular accidents, especially in the elderly, where their prevalence tends to be similar to that of COPD [8,20]. Independently of the alleged role by inhaled β2-adrenergic agonist in enhancing cardiac comorbidities and death, both in COPD and asthma [34], an endothelial pathogeny linked to bronchial inflammation has been invoked as possibly responsible for the cardiovascular diseases associated with or coincident with asthma. Some studies have correlated bronchial inflammation with the production of C reactive protein (CRP) in the vascular endothelium of the bronchial tubes, through the stimulation of IL-6. CRP, in turn, has been implicated directly in atherosclerosis and its complications [35,36]. This would result in a lesion of the vascular endothelium and, in consequence, the development of cardiovascular disease. A similar mechanism has also been suggested in the pathogeny of endothelial lesions of COPD [37]. On the other hand, the possible role of the cysteinyl leukotrienes has been proposed; in addition to constituting one of the principal inflammatory mediators of asthma, these molecules may cause endothelial lesion or dysfunction [38]. Particularly in women, in whom they are promoted by circulating estrogens, cysteinyl leukotrienes could contribute to the greater incidence of cardiovascular diseases observed in these patients. Hence those patients with active asthmatic inflammatory disease, who therefore produce more leukotrienes, would have a greater risk of suffering cardiovascular diseases [39,40]. The hypothesis is coherent with previous observations particularly in asthmatic women, in whom the association with suffering or dying from cardiovascular, and especially cerebrovascular diseases, appears more consistent [17,26,27]. Our results, 78% of female asthma deaths who presented an exceptionally aggressive asthmatic disease support that hypothesis. Of these, 49% were suffering a severe or moderate asthma, 85.5% were not receiving sufficient monitoring of the disease and the mean of previous hospital admissions for asthma was two. All of them were largely modifiable factors. In addition, they had a greater proportion of obese patients (32.8%) and overall a significantly greater BMI (mean of 27.6 kg/cm^2^), in comparison with the two reference groups as suggested elsewhere [41], it could contribute to a greater risk of accompanying vascular disease. However, the multivariate study carried out did not confirm the association between asthma and death from a cardiovascular disease (OR 0.95; p = 0.9809), nor with any of its variants considered in the study. Perhaps a greater sample size could have identified a statistical association, for example, with death due to heart failure which showed results close to significance, in agreement with Proser et al [42], reporting a strong association of both (OR 4.62).

Although the study is subject to the customary methodological limitations due to the retrospective collection of information from patients clinical records, we consider that, with our choice of study design with two reference populations this potential limitation was lessened. Data quality on causes of death is greater when obtained from hospital charts in comparison when obtained via death certificates of deaths occurring outside the hospital context, in which the nature of the cause of death can often be questionable. Perhaps one strength here is the rigor employed in the assumption of the diagnosis of asthma in all deaths analyzed. Anyway, our conclusions must be tempered by the large number of statistical tests carried out.

To conclude, the most common causes of death of asthmatics in our study were those related to cardiovascular diseases and, unlike COPD patients, there were significantly fewer deaths due to exacerbation and lung cancer. These findings are relevant, as mortality in asthma has been analyzed only partially and mostly within subgroups. We did not observed an association between suffering asthma and death from a cardiovascular disease, but similar studies need to be conducted with larger samples of asthmatics, in which causes of death should be analyzed rigorously. Until such information be available, the present study could provide arguments for adopting a more proactive attitude in routine clinical practice, towards the prevention and treatment of cardiovascular diseases in patients with asthma, particularly in those of advanced age.

## Competing interests

The authors declare that they have no competing interest.

## Authors’ contributions

The work presented here was carried out in collaboration between all authors. GS, VP, JBS defined the research theme and wrote the paper. GS, VS, designed methods, analyzed the data, interpreted the results. CC-L, CA, RV-O, JS, AB-Q and AP co-designed the dispersal and co-worked on associated data collection and interpretation. All authors have contributed to, seen and approved the manuscript.

## Pre-publication history

The pre-publication history for this paper can be accessed here:

http://www.biomedcentral.com/1471-2466/13/73/prepub
